# Cleidocranial dysplasia and novel *RUNX2* variants: dental, craniofacial, and osseous manifestations

**DOI:** 10.1590/1678-7757-2022-0028

**Published:** 2022-06-06

**Authors:** Sermporn THAWEESAPPHITHAK, Jirawat SAENGSIN, Wuttichart KAMOLVISIT, Thanakorn THEERAPANON, Thantrira PORNTAVEETUS, Vorasuk SHOTELERSUK

**Affiliations:** 1 Chulalongkorn University Faculty of Dentistry Department of Physiology Bangkok Thailand Chulalongkorn University , Faculty of Dentistry , Department of Physiology , Center of Excellence in Genomics and Precision Dentistry, Bangkok , Thailand .; 2 Chiang Mai University Faculty of Medicine Department of Orthopedic Surgery Chiang Mai Thailand Chiang Mai University , Faculty of Medicine , Department of Orthopedic Surgery , Chiang Mai , Thailand .; 3 Chulalongkorn University Faculty of Medicine Department of Pediatrics, Medical Genomics Cluster Bangkok Thailand Chulalongkorn University , Faculty of Medicine , Department of Pediatrics, Medical Genomics Cluster , Center of Excellence for Medical Genomics, Bangkok , Thailand .; 4 King Chulalongkorn Memorial Hospital Excellence Center for Genomics and Precision Medicine Bangkok Thailand The Thai Red Cross Society, King Chulalongkorn Memorial Hospital , Excellence Center for Genomics and Precision Medicine , Bangkok , Thailand .

**Keywords:** Cranial sutures, Malocclusion, Tooth, Supernumerary, Tooth, Unerupted, Wormian bones, Wide fontanelle

## Abstract

Cleidocranial dysplasia (CCD) is a skeletal disorder affecting cranial sutures, teeth, and clavicles, and is associated with the *RUNX2* mutations. Although numerous patients have been described, a direct genotype–phenotype correlation for *RUNX2* has been difficult to establish. Further cases must be studied to understand the clinical and genetic spectra of CCD.

**Objectives:**

To characterize detailed phenotypes and identify variants causing CCD in five unrelated patients and their family members.

**Methodology:**

Clinical and radiographic examinations were performed. Genetic variants were identified by exome and Sanger sequencing, data were analyzed by bioinformatics tools.

**Results:**

Three cases were sporadic and two were familial. Exome sequencing successfully detected the heterozygous pathogenic *RUNX2* variants in all affected individuals. Three were novel, comprising a frameshift c.739delA (p.(Ser247Valfs*)) in exon 6 (Patient-1), a nonsense c.901C>T (p.(Gln301*)) in exon 7 (Patient-2 and affected mother), and a nonsense c.1081C>T (p.(Gln361*)) in exon 8 (Patient-3). Two previously reported variants were missense: the c.673C>T (p.(Arg225Trp)) (Patient-4) and c.674G>A (p.(Arg225Gln)) (Patient-5) in exon 5 within the Runt homology domain. Patient-1, Patient-2, and Patient-4 with permanent dentition had thirty, nineteen, and twenty unerupted teeth, respectively; whereas Patient-3 and Patient-5, with deciduous dentition, had normally developed teeth. All patients exhibited typical CCD features, but the following uncommon/unreported phenotypes were observed: left fourth ray brachymetatarsia (Patient-1), normal clavicles (Patient-2 and affected mother), phalangeal malformations (Patient-3), and normal primary dentition (Patient-3, Patient-5).

**Conclusions:**

The study shows that exome sequencing is effective to detect mutation across ethnics. The two p.Arg225 variants confirm that the Runt homology domain is vital for *RUNX2* function. Here, we report a new CCD feature, unilateral brachymetatarsia, and three novel truncating variants, expanding the phenotypic and genotypic spectra of *RUNX2* , as well as show that the CCD patients can have normal deciduous teeth, but must be monitored for permanent teeth anomalies.

## Introduction

Cleidocranial dysplasia (CCD, MIM #119600) is a congenital disorder that affects bone growth and teeth formation. ^1^ The incidence of CCD is about one in a million. ^2 , 3^ CCD is also known as Scheuthauer-Marie-Sainton syndrome, cleidocranial dysostosis, oste-odental dysplasia, generalized dysostosis, mutational dysostosis, and cleidocranial-pubic dysostosis. ^1 , 4^ The main clinical features of CCD are hypoplastic or aplastic clavicles, defective ossification of anterior fontanelle, and dental anomalies (retention teeth, supernumerary teeth, impacted teeth, and cysts). ^1 , 3^ The unique facial appearances of CCD are prominent forehead, midfacial hypoplasia, and hypertelorism. Other bone deformities include wide pelvic joint, coxa vara (less than 120 degrees angle between the femoral head and shaft), coxa valga (increased angle between the femoral neck and shaft), genu valgum (knock-knee deformity), delayed growth of the pubic bone, merging failure of the lower jaw, scoliosis, and brachydactyly. ^3 , 5^ Treatment for CCD depends on the symptoms. The patient may undergo several orthopedic and facial reconstructive surgery to increase satisfaction and function. ^6^ Moreover, Orthodontics and oral surgery treatment are necessary to remove extra teeth and guide the eruption of affected teeth. ^7^

Previous studies have reported that the human CCD is caused by various variants in the *RUNX2* gene. ^3 , 8 , 9^ The human runt-related transcription factor 2 gene ( *RUNX2* , OMIM *60021) consists of 9 exons, but only 8 exons encode the 521-amino acid RUNX2 protein. The RUNX2 comprises a poly-glutamine and poly-alanine (QA, amino acids 49-89) repeat region, runt homologous domain (RHD amino acids 101-226), nuclear localization signal (NLS, amino acids 227-235), proline–serine–threonine (PST)-rich region (amino acids 236-516), nuclear matrix targeting signal (NMTS, amino acids 390-427), and VWRPY pentapeptide sequence (amino acids 517-521). ^10 , 11^ Both the QA and PST regions act as the transactivation regions. The RHD is highly conserved and plays a role in DNA binding. ^10^ The VWRPY pentapeptide sequence functions as a transcriptional repression region. ^10^

RUNX2 is a transcription factor that either activates or inhibits the gene expression by regulating transcription through binding specific DNA sequences or interacting with transcriptional co-inducers and co-depressors to regulate osteogenesis. ^12^ In humans, the heterozygous loss-of-function variants in *RUNX2* are the major cause of CCD (60-70% of patients). However, the genetic conditions in the remaining 30-40% of the cases are still unknown. Direct genotype–phenotype correlation for RUNX2 has been difficult to establish due to variable phenotypic penetrance of the variants. Most variants in the RHD domain result in a classical CCD phenotype. ^13^ However, some exceptions like the families with p.Thr200Ala exhibited significant intrafamilial variability: two of four children showed classic CCD, while the father, who also possessed the variant, only had dental anomalies. ^14^ Diverse intra- and inter-familial genotype-phenotype correlations have been reported. For example, in a family with c.203delAinsCG, mild intra-familial genotype-phenotype were observed, including bell-shaped rib cage, hypoplastic iliac wing, and hypoplastic femoral head; in a family with c.614C>G, significant intra-familial correlation were observed, including head and neck findings, delayed tooth eruption, hypoplastic clavicle, and short stature; and in patients with c.1281delC, no inter- or intra-familial correlation was found. ^15^ In a study of 42 unrelated CCD patients, there was no phenotypic difference between patients with deletions or frameshifts and those with other intragenic variants in RUNX2, ^16^ whereas another study, with 24 unrelated Japanese patients, observed short stature to be much milder in the patients with the intact RHD domain than in those without. ^9^ Despite having more than 180 different pathogenic variants identified, the genotype-phenotype correlation of the syndrome is still unclear. ^15 , 16^ The identification of novel variants and phenotypes can provide more understanding about CCD and how each variant contributes to different expressivity.

Although numerous patients have been described, more patients need to be reported for a better understanding of its clinical and genetic spectra. Herein, we describe the clinical and molecular characteristics of five unrelated Thai CCD patients. Exome sequencing successfully identified causative variants in *RUNX2* in all five cases. Three were novel pathogenic variants, expanding the genotypic spectrum of *RUNX2* .

## Methodology

### Subject Enrollment

Five unrelated Thai patients clinically diagnosed with CCD (Patient-1 to Patient-5) and six additional family members of Patients-2, -3, and -4 were recruited. Thorough investigation and blood collection were performed after receiving the written informed consent from all subjects or their legal guardians. The study was approved by the Human Research Ethics Committee (HREC-DCU 2021-030, Date of approval: 9 July 2021) and complied with the Declaration of Helsinki. The clinical diagnostic criteria of CCD involve (a) hypoplasia or aplasia of the clavicles, (b) prominent forehead, enlargement of the fontanelles or delayed closure of the anterior fontanelle, and/or (c) retention of primary teeth, failure of secondary teeth, and supernumerary teeth. ^1 , 5 , 17^ Tooth number was used according to the FDI World Dental Federation notation system or ISO 3950 Dentistry – Designation system for teeth and areas of the oral cavity (https://www.iso.org/standard/68292.html).

### Genetic Analyses

Genomic DNA extracted from 3 ml of peripheral blood leukocytes of the probands, their available family members, and from healthy individuals (as control) was subjected to variant analysis using exome or Sanger sequencing. For exome sequencing, genomic DNA was captured using a SureSelect Human All Exon version 4 kit (Agilent Technologies, Santa Clara, CA, United States) and sequenced using Hiseq 2000 Sequencer (Macrogen, Seoul, South Korea). The sequence reads were aligned to the human genome reference sequence (University of California Santa Cruz (UCSC) hg19) using the Burrows-Wheeler Alignment software (http://bio-bwa.sourceforge.net/). Downstream processing was performed with SAMtools (http://samtools.sourceforge.net/) and annotated by the dbSNP and 1000 Genomes. Subsequently, the variants were filtered out if their frequency were higher than 1% in the 1000 Genomes Project, Genome Aggregation Database (gnomAD), and our in-house database of 2,166 Thai exomes. The causal variants Identified in *RUNX2* (NM_001024630.4, NP_001019801.3) were confirmed by Sanger sequencing (Supplementary Table 1). Prediction software, PolyPhen-2, SIFT, and MutationTaster, were used to analyze the potential pathogenicity of the variants based on the possible impact structure and function of the protein. The American College of Medical Genetics and Genomics and the Association for Molecular Pathology (ACMG-AMP) guideline was used to classify the variants. ^18^ The variants were identified as novel if they were not published in the ClinVar-NCBI (https://www.ncbi.nlm.nih.gov/clinvar) and the gnomAD (https://gnomad.broadinstitute.org/).

**Table 1:** Phenotypes of the CCD patients with different *RUNX2* variants

Characteristics	1-I:2	2-II:1	3-II:1	4-II:2	5-II:1
Genetic variants	c.739delA (p.(Ser247Valfs*3))	c.901C>T (p.(Gln301*))	c.1081C>T (p.(Gln361*))	c.673C>T (p.(Arg225Trp))	c.674G>A (p.(Arg225Gln))
Age (years)	28	21	6	20	3
Gender	Female	Male	Female	Female	Male
Clavicles	Hypoplasia	Normal	Aplasia	Aplasia	Hypoplasia
Abnormal facility in opposing the shoulders	Y	N	Y	Y	Y
**Dental features**					
Dentition	Permanent	Permanent	Deciduous	Permanent	Deciduous
Unerupted permanent teeth	30 teeth	19 teeth	NA	20 teeth	NA
Unerupted supernumerary teeth	5 teeth	7 teeth	2 teeth (deciduous upper incisors)	8 teeth	N
Malocclusion	Y	Y	Y	Y	Y
Crossbite	Y	Y	Y	Y	Y
Retention of deciduous teeth	Y	Y	N	Y	N
Delayed eruption of deciduous teeth	NA	NA	NA	NA	Y
**Craniofacial features**					
Open/delayed closure of fontanelle	Y	Y (minimal)	Y	Y	Y
Wormian bones	Y	Y	Y	Y	Y
Frontal bossing	Y	Y	Y	Y	Y
Parietal bossing	N	Y	Y	N	Y
Metopic depression	Y	Y	Y	Y	Y
Maxillary hypoplasia	Y	Y	N	Y	Y
**Other features**					
Hands	Brachydactyly	N	Partial pseudoepiphysis of 2 ^nd^ and 5 ^th^ proximal metacarpal bone, shortening of 2 ^nd^ and 5 ^th^ middle phalanges, fusion of 1 ^st^ distal phalanx	N	N
Feet	Fourth-ray brachymetatarsia of the left foot	N	N	N	N

Y, present; N, not present; NA, not available

## Results

### Exome sequencing successfully identified the heterozygous variants in *RUNX2* in all affected cases.

**Figure 1:**
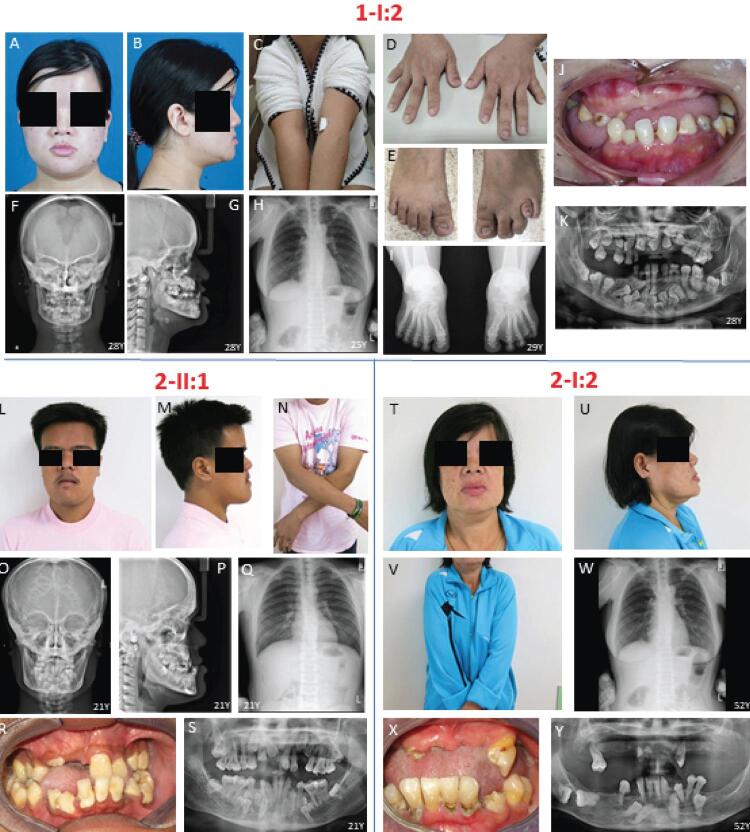
Clinical and radiographic features of 1-I:2, 2-II:1, and 2-I:2. (A-K) 1-I:2 shows typical CCD craniofacial features, narrow shoulder, and brachydactyly and short fourth toe of the left foot. Radiographs shows hypoplasia of the lateral part of both clavicles, widened cranial sutures, Wormian bones, fourth-ray brachymetatarsia of the left foot, unerupted teeth, and supernumerary teeth. (L-S) 2-II:1 shows normal clavicles, CCD facial features with asymmetry, widened cranial sutures, Wormian bones, spina bifida in the lower cervical spine, scoliosis of the lower thoracic spine, supernumerary teeth. (T-Y) 2-I:2 has unerupted teeth and normal clavicles

**Figure 3:**
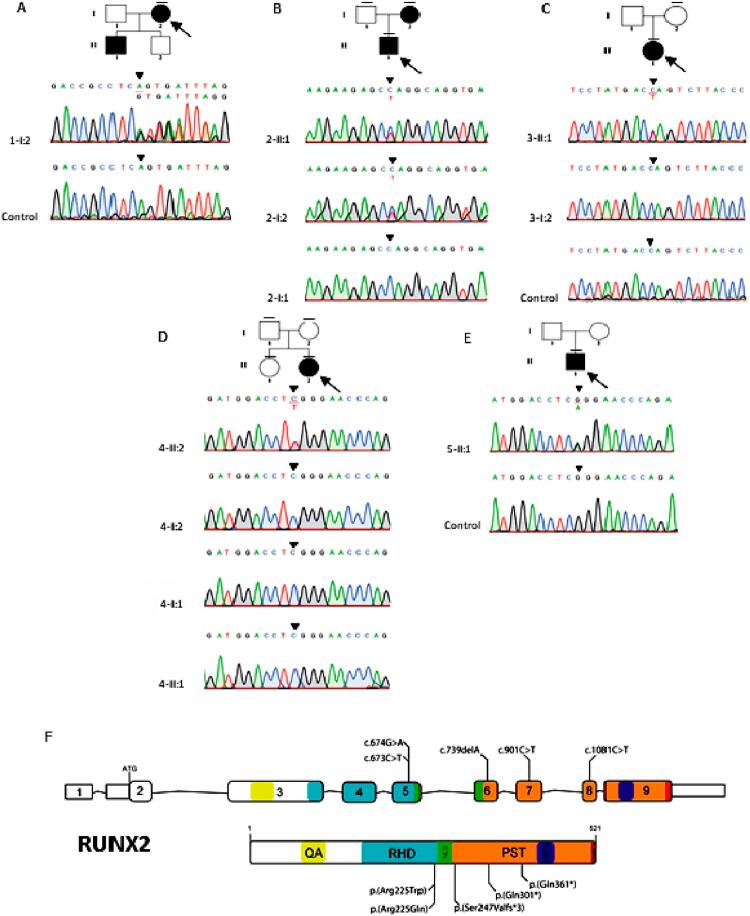
Pedigrees and genetic variants of Family 1-5. (A) 1-I:2 possesses the frameshift variant, c.739delA (p.(Ser247Valfs*3)), in RUNX2. (B) 2-II:1 and 2-I:2 possesses the nonsense variant, c.901C>T (p.(Gln301*)), in RUNX2. The variant was not identified in 2-I:1. (C) 3-II:1 has the nonsense variant, c.1081C>T (p.(Gln361*)), in RUNX2. (D) 4-II:2 has the missense variant, c.673C>T (p.(Arg225Trp)), in RUNX2. 4-I:1, 4-I:2 and 4-II:1 did not have the variants. (E) 5-II:1 possesses the missense variant, c.674G>A (p.(Arg225Gln)) in RUNX2. The arrow indicates the proband. The dash above each symbol indicates the subject with genetic test. (F) Schematic diagrams of RUNX2 gene and protein showed the locations of RUNX2 variants identified in this study. QA, poly-glutamine and poly-alanine repeat region; RHD, runt homologous domain; NLS, nuclear localization signal; PST, proline-serine-threonine-rich region; NMST, nuclear matrix targeting sequence; VWRPY, pentapeptide sequence

Patient-1 (1-I:2), a 28-year-old female, complained of missing teeth and chewing difficulty. She had short stature, narrow and down-sloping shoulders, metopic depression, hypertelorism, prominent orbital ridges, and protruding chin. Brachydactyly and short fourth toe of the left foot were also observed ( Figure 1A - E ). Skull radiographs showed widened cranial sutures, Wormian bones, frontal and parietal bossing, concave frontonasal suture, maxillary hypoplasia, and mandibular prognathism. Anteroposterior thoracolumbar spine radiograph showed hypoplasia of the lateral part of both clavicles, spina bifida in the lower cervical spine, and narrow thorax ( Figure 1F – H ). Anteroposterior foot radiograph showed fourth-ray brachymetatarsia of the left foot ( Figure 1I ). Oral examination showed 9 erupted permanent teeth (16, 14, 25, 26, 27, 36, 35, 31, 41), 6 retained deciduous teeth (52, 53, 73, 74, 82, 83), crossbite, class III malocclusion, hypoplastic mandibular edentulous ridge, multiple caries, erosion, and gingival inflammation ( Figure 1J ). Panoramic radiograph showed 30 unerupted teeth including 5 supernumerary teeth ( Figure 1K ). Exome sequencing showed that 1-I:2 was heterozygous for a novel frameshift variant, c.739delA (p.(Ser247Valfs*3)), in exon 6 of *RUNX2* (SCV001763563) ( Figure 3A , F ).

Patient-2 (2-II:1), a 21-year-old male, was referred for orthognathic surgery due to chewing difficulty. Clinical examination showed that the patient had a dolichofacial shape, facial asymmetry (chin shift to the right side), metopic depression, and protruding chin ( Figure 1L - N ). Skull radiographs showed widened cranial sutures, Wormian bones, frontal and parietal bossing, maxillary hypoplasia, and skeletal class III malocclusion ( Figure 1O – P ). Chest radiograph exhibited spina bifida in the lower cervical spine and scoliosis of the lower thoracic spine ( Figure 1Q ). Oral examination showed 20 erupted permanent teeth including 1 partially erupted, mesiodens, retained deciduous lower right incisor, narrow and high-arched palate, crossbite, dental caries, and periodontitis ( Figure 1R ). Panoramic radiograph showed 19 unerupted teeth, comprising teeth 11, 13, 18, 23, 24, 28, 33, 35, 38, 43, 45, 48; six supernumerary teeth in the mandible; and a mesiodens in the maxilla ( Figure 1S ). The upper left first molar developed pulp necrosis with asymptomatic apical periodontitis. Patient-2’s mother (2-I:2) presented facial asymmetry, protruding chin, unerupted permanent teeth, class III malocclusion, and gingival inflammation ( Figure 1T – Y ). 2-II:1 and 2-I:2 did not have hypoplastic clavicles and they were unable to oppose the shoulders at the midline. Both possessed a novel heterozygous nonsense variant, c.901C>T (p.(Gln301*)), in exon 7 of *RUNX2* (SCV001763562) ( Figure 3B , F ).

**Figure 2:**
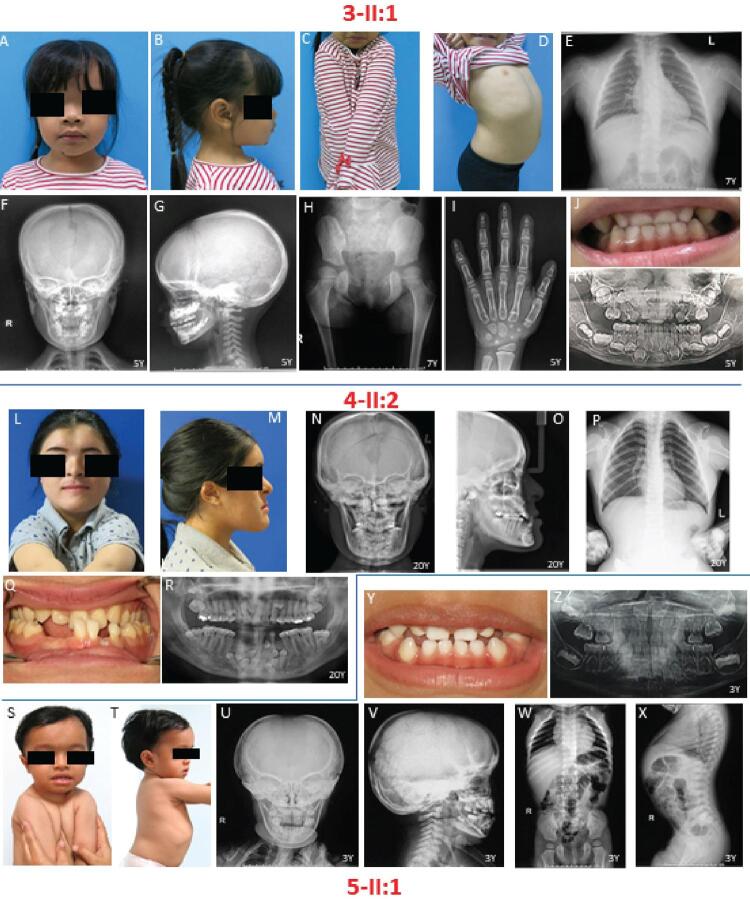
Clinical and radiographic features of 3-II:1, 4-II:2, and 5-II:1. (A-K) 3-II:1 shows macrocephaly and lordosis. Radiographs shows aplastic clavicles, a “chef’s hat” appearance of the femoral head, partial pseudoepiphysis of 2 nd and 5 th proximal metacarpal bone, shortening of 2 nd and 5 th middle phalanges, fusion of 1st distal phalanx, and supernumerary upper incisors. (L-R) 4-II:2 has square-shaped face, aplastic clavicle, unerupted teeth, and supernumerary teeth. (S-Z) 5-II:1 shows bilateral deformity and pseudarthrosis of clavicles, lordosis at lumbar spine, lack of ossification of pubic bones, and widening of the pubic symphysis

Patient-3 (3-II:1), a 6-year-old girl, presented short stature (height 101.5 cm, <3 ^rd^ percentile), macrocephaly (OFC 54 cm, >97 ^th^ percentile), metopic depression, saddle nose, hypertelorism, small shoulders that could be opposed at the midline, and lordosis ( Figure 2A – D ). Chest radiograph disclosed absence of both clavicles ( Figure 2E ). Skull radiographs showed unclosed fontanelle, frontal and parietal bossing, persistent metopic suture, multiple Wormian bones along the bilateral lambdoid and posterior aspect of squamosal sutures, and prominent adenoid tissue and bilateral palatine tonsils. Maxillary hypoplasia was not evident ( Figure 2F – G ). Anteroposterior hip radiograph showed a lack of ossification of pubic bones with the resultant apparent widening of the pubic symphysis and a characteristic “chef’s hat” appearance of the femoral head ( Figure 2H ). Hand radiograph exhibited partial pseudoepiphysis of 2 ^nd^ and 5 ^th^ proximal metacarpal bones, shortening of 2 ^nd^ and 5 ^th^ middle phalanges, and fusion of 1 ^st^ distal phalanx ( Figure 2I ). Oral examination revealed deciduous dentition, crossbite, and two supernumerary teeth in the upper incisor area ( Figure 2J , K ). 3-II:1 at 5 years 10 months old had the bone age of 4 years and 2 months according to Greulich and Pyle ^19^ (1959). A novel heterozygous nonsense variant, c.1081C>T (p.(Gln361*)), in exon 8 of *RUNX2* (SCV001763561) was detected ( Figure 3C , F ). The mother was unaffected. The father was reported not to be affected.

Patient-4 (4-II:2), a 20-year-old female, had a chief complaint of prominent chin and tooth missing. The patient presented a square-shaped face, metopic depression, and a slant and narrow shoulder that can be opposed at the midline ( Figure 2L - M ). Skull radiographs showed widened cranial sutures, frontal and parietal bossing, Wormian bones, maxillary hypoplasia, and class III skeletal malocclusions ( Figure 2N – O ). Chest radiograph showed aplastic clavicle and narrow thorax ( Figure 2P ). Orodental features include crossbite, malocclusion, 20 erupted permanent teeth, 2 retained deciduous teeth, 20 unerupted teeth including 8 supernumerary teeth (3 in the upper and 5 in the lower anterior regions) ( Figure 2Q, R ). The lower right first premolar developed pulp necrosis and asymptomatic apical periodontitis. The patient possessed the *de novo* heterozygous missense variant, c.673C>T (p.(Arg225Trp)), in exon 5 of *RUNX2* (SCV001780129, dbSNP rs104893992) ( Figure 3D , F ). ^16^ Her parents were unaffected and did not carry the missense. The variant was previously reported in the familial and sporadic cases with CCD. ^13 , 16 , 20^

Patient-5 (5-II:1), a 3-year-old boy, was referred for genetic analysis due to congenital malformations of skull and face bones, deformed clavicles, and a history of bilateral clavicular fractures. At 4 months old, his whole-body bone mineral density (BMD) was 0.267 g/cm ^2^ within normal range (0.25 + 0.04 g/cm ^2^ ); total body less head BMD was 0.267 g/cm ^2^ ; lumbar spine BMD was 0.142 g/cm ^2^ ; and whole-body mineral content was 163.14 g. At age 3, his height was 86.2 cm (<3 ^rd^ percentile). Examinations showed that 5-II:1 had macrocephaly (OCF 52 cm, >97 ^th^ percentile), metopic depression, narrow sloping shoulders opposing at the midline, and lordosis ( Figure 2S-T ). Skull radiographs showed unclosed fontanelle, widened skull sutures, multiple Wormian bones in parietal and occipital bones, frontal and parietal bossing, maxillary hypoplasia, and crossbite. The radiograph showed bilateral deformity and pseudarthrosis of both clavicles, lack of ossification of pubic bones with apparent widening of the pubic symphysis, and increased lordosis at the lumbar spine ( Figure 2W – X ). The patient had crossbite of deciduous teeth and a history of delayed tooth eruption. Other dental anomalies were not observed ( Figure 2Y - Z ). His bone age at 3 years corresponded with his chronological age. ^19^ The patient possessed the *de novo* heterozygous missense variant, c.674G>A (p.(Arg225Gln)), in exon 5 of *RUNX2* (SCV002102512, dbSNP rs104893991) ( Figure 3E , F ). ^16^ This variant was not detected in the parents. It was previously reported in the familial and sporadic CCD cases. ^13 , 16 , 20^

Phenotypically, all patients in this study shared typical features of CCD consisting of frontal and parietal bossing, metopic depression, unclosed fontanelle, and Wormian bones. Except for 2-II:1 and 2-I:2, all patients had hypoplastic/aplastic clavicles and abnormal facility in opposing the shoulders. 1-I:2, 2-II:1, 2-I:2, and 4-II:2 who had the permanent dentition showed multiple dental anomalies including clinically missing teeth, unerupted teeth, supernumerary teeth, and skeletal class III malocclusion. In contrast, 3-II:1 and 5-II:1 showed all deciduous teeth erupted and only crossbite. Table 1 shows phenotypic data. Table 2 shows genetic variants, bioinformatics data, and variant interpretation according to the American College of Medical Genetics and Genomics (ACMG) guideline. ^18^

**Table 2:** The heterozygous *RUNX2* variants identified in the CCD patients

Patient	Inheritance	Variants	Clinvar accession number	Zygosity	Variant type	Exon	Domain/Region	ACMG-AMP guideline	Status
1-I:2	Familial	c.739delA (p.(Ser247Valfs*3))	SCV001763563	Het	Frameshift	6	PST	Pathogenic	Novel
2-II:1 & 2-I:2	Familial	c.901C>T (p.(Gln301*))	SCV001763562	Het	Nonsense	7	PST	Pathogenic	Novel
3-II:1	NA	c.1081C>T (p.(Gln361*))	SCV001763561	Het	Nonsense	8	PST	Pathogenic	Novel
4-II:2	Sporadic	c.673C>T (p.(Arg225Trp))	SCV001780129	Het	Missense	5	RHD	Pathogenic	Previously reported†
5-II:1	Sporadic	c.674G>A (p.(Arg225Gln))	SCV002102512	Het	Missense	5	RHD	Pathogenic	Previously reported‡

M, male; F, female; NA, not available; RHD, Runt homologous domain; PST, proline-serine-threonine region; Del, deleterious; Het, heterozygous

ACMG-AMP, The American College of Medical Genetics and Genomics and the Association for Molecular Pathology

†1-10; ‡2, 5, 6, 8, 11-20 (References are provided in the supplementary figure 1)

## Discussion

Approximately two-thirds of CCD cases have been found to harbor pathogenic variants in *RUNX2* and with heterogeneous phenotypic penetrance. ^3 , 15^ In this study, we reported five index patients with five different *RUNX2* variants who had classic craniofacial features of CCD with clinical heterogeneity.

Three truncating variants identified in the PST region were novel. The variants in the PST were shown to produce non-functional truncated proteins and impair the transactivation activities of RUNX2 on osteocalcin promoter, indicating that loss of function in RUNX2 is responsible for CCD. ^21^ Most of the missense variants have been observed in the RHD domain. ^15 , 16^ Consistently, the other two missense variants (c.673C>T and c.674G>A) were detected in the hotspot RHD domain and previously reported in many cases with typical CCD features. ^8 , 16 , 21 , 22^ Although these two variants alter the same amino acid p.Arg225, different clinical features were observed in patients 4-II:2 and 5-II:1. Patient 4-II:2 had aplastic clavicles and no parietal bossing, whereas 5-II:1 had deformity and pseudarthrosis of clavicles and lordosis. Functional analyses showed that the variants at Arg225 markedly reduced transactivation activities and interfered with nuclear accumulation of the RUNX2 protein, resulting in haploinsufficiency of the protein. ^9 , 16 , 22^

Normal clavicles were observed in Patients 2-II:1 and 2-I:2, who possessed the novel p.Gln301* variant in the PST region. They also shared similar craniofacial features, including facial asymmetry, class III malocclusion, and unerupted teeth, suggesting intra-familial genotype-phenotype correlation. Normal clavicles were previously reported in CCD cases with different *RUNX2* variants, including the missense in the RHD domain and the truncating variants in the N-terminal, QA, and PST regions. ^3 , 15 , 23 , 24^ A correlation between dental abnormalities and location of *RUNX2* variants was demonstrated in a group of five patients having normal clavicles. The nonsense variants in the QA region presented milder dental phenotypes than those with missense variants in the RHD domain. ^3 , 23^ With variants in the PST region and normal clavicles, previous CCD cases were reported with multiple supernumerary teeth. ^25 , 26^ Similarly, supernumerary teeth were found in patients 2-II:1 and 2-I:2. Further studies are needed to confirm this genotype-phenotype correlation.

During tooth development, *RUNX2* is expressed from early stage to the formation of roots and periodontium. ^27^ It plays a role in osteoblast and odontoblast differentiation, alveolar bone remodeling essential for tooth eruption, and the maintenance of the periodontal ligament. ^28^ In *RUNX2* knockout mice, molar tooth development arrested at the late bud stage. ^29^ Interestingly, both *Runx2* ^–/–^ and *Runx2* ^-^ mice developed lingual buds in front of the upper molars more than in wild-type mice, representing the extension of dental lamina for successional teeth. ^30^ It was suggested that *Runx2* may have different effects at different stages of tooth development. Accordingly, the development of permanent dentition in CCD patients is severely disturbed while the primary dentition is rarely affected. ^1 , 28 , 31^ Patients 1-I:2, 2-II:1, 2-I:2, and 4-II:2 with permanent dentition, showed several dental anomalies, such as malocclusion, clinically missing of permanent teeth, unerupted teeth, and supernumerary teeth; whereas patients 3-II:2 and 5-II:1, with primary dentition, showed no remarkable tooth abnormalities. It may also be assumed that the effects of RUNX2 haploinsufficiency on the development of the primary dentition may be too subtle to be detected clinically. ^28^

Apart from typical CCD features, 1-I:2 also presented with brachydactyly and fourth-ray brachymetatarsia of the left foot and 3-II:1 had partial pseudoepiphysis of the 2 ^nd^ and 5 ^th^ proximal metacarpal bone, shortening of the 2 ^nd^ and 5 ^th^ middle phalanges, and fusion of the 1 ^st^ distal phalanx. Hand abnormalities, including brachydactyly, tapering fingers, and short and broad thumbs, are frequently observed in CCD patients. ^17 , 32 - 34^ A decrease or loss-of-function of *RUNX2* is associated with CCD; while gain-of-function due to intragenic duplications is related to metaphyseal dysplasia with maxillary hypoplasia and brachydactyly (MDMHB, MIM #156510). ^35 , 36^ These suggest that fine-tuning the expression level of *RUNX2* is important for normal development of the phalanges or metacarpals. There have been only few clinical case reports describing brachymetatarsia in CCD cases, but without genetic variant identification. ^37 - 39^ Our findings could lead to genotype-phenotype correlation in the future.

## Conclusions

This study showed that *RUNX2* is the most common causative gene of CCD in this small sample of five patients. Here, we report three novel *RUNX2* variants expanding the genotypic spectrum of CCD and confirm that the *RUNX2* missense variants associated with typical CCD features are commonly observed in the RHD domain. Additionally, we demonstrated that a patient with the novel frameshift variant, c.739delA (p.(Ser247Valfs*3)), in *RUNX2* had brachymetatarsia, substantiating it as a clinical feature of CCD.

## Supplementary Material





## References

[1] - Mundlos S . Cleidocranial dysplasia: clinical and molecular genetics . J Med Genet . 1999 ; 36 : 177 - 82 . PMC1734317 10204840

[2] - Machol K , Mendoza-Londono R , Lee B . Cleidocranial dysplasia spectrum disorder . In: Adam MP , Ardinger HH , Pagon RA , Wallace SE , Bean LJ , Gripp KW , et al ., editors . GeneReviews® [ Internet ]. Seattle (WA ): University of Washington, Seattle ; 2006 [ Updated 2017 Nov 16 ]. Available from: https://www.ncbi.nlm.nih.gov/books/NBK1513/20301686

[3] - Baumert U , Golan I , Redlich M , Aknin JJ , Muessig D . Cleidocranial dysplasia: molecular genetic analysis and phenotypic-based description of a Middle European patient group . Am J Med Genet A . 2005 ; 139A ( 2 ): 78 - 85 . doi: 10.1002/ajmg.a.30927 10.1002/ajmg.a.30927 16222673

[4] - Kuruvila VE , Bilahari N , Attokkaran G , Kumari B . Scheuthauer-Marie-Sainton syndrome . Contemp Clin Dent . 2012 ; 3 ( 3 ): 338 - 40 . doi: 10.4103/0976-237X.103632 10.4103/0976-237X.103632 PMC3532802 23293495

[5] - Farrow E , Nicot R , Wiss A , Laborde A , Ferri J . Cleidocranial dysplasia: a review of clinical, radiological, genetic implications and a guidelines proposal . J Craniofac Surg . 2018 ; 29 ( 2 ): 382 - 9 . doi: 10.1097/SCS.0000000000004200 10.1097/SCS.0000000000004200 29189406

[6] - Li J , Shen J , Xu J , Weng L , Pan J , Lin J . The treatment strategy of cleidocranial dysplasia: combined orthodontic and orthognathic treatment . J Craniofac Surg . 2019 ; 30 ( 6 ): 1767 - 71 . doi: 10.1097/SCS.0000000000005372 10.1097/SCS.0000000000005372 30950953

[7] - Li ZJ , Wang JY , Gao MF , Wu DL , Chang X . Orthodontic treatment of a patient with cleidocranial dysplasia: a case report . Exp Ther Med . 2016 ; 12 ( 2 ): 690 - 4 . doi: 10.3892/etm.2016.3430 10.3892/etm.2016.3430 PMC4950898 27446262

[8] - Zhang X , Liu Y , Wang X , Sun X , Zhang C , Zheng S . Analysis of novel RUNX2 mutations in Chinese patients with cleidocranial dysplasia . PLoS One . 2017 ; 12 ( 7 ): e0181653 . doi: 10.1371/journal.pone.0181653 10.1371/journal.pone.0181653 PMC5524338 28738062

[9] - Yoshida T , Kanegane H , Osato M , Yanagida M , Miyawaki T , Ito Y , et al . Functional analysis of RUNX2 mutations in Japanese patients with cleidocranial dysplasia demonstrates novel genotype-phenotype correlations . Am J Hum Genet . 2002 ; 71 ( 4 ): 724 - 38 . doi: 10.1086/342717 10.1086/342717 PMC378531 12196916

[10] - Thirunavukkarasu K , Mahajan M , McLarren KW , Stifani S , Karsenty G . Two domains unique to osteoblast-specific transcription factor Osf2/Cbfa1 contribute to its transactivation function and its inability to heterodimerize with Cbfbeta . Mol Cell Biol . 1998 ; 18 ( 7 ): 4197 - 208 . doi: 10.1128/MCB.18.7.4197 10.1128/mcb.18.7.4197 PMC109004 9632804

[11] - Zaidi SK , Javed A , Choi JY , van Wijnen AJ , Stein JL , Lian JB , et al . A specific targeting signal directs Runx2/Cbfa1 to subnuclear domains and contributes to transactivation of the osteocalcin gene . J Cell Sci . 2001 ; 114 ( Pt 17 ): 3093 - 102 . doi: 10.1242/jcs.114.17.3093 10.1242/jcs.114.17.3093 11590236

[12] - Liu TM , Lee EH . Transcriptional regulatory cascades in Runx2-dependent bone development . Tissue Eng Part B Rev . 2013 ; 19 ( 3 ): 254 - 63 . doi: 10.1089/ten.TEB.2012.0527 10.1089/ten.teb.2012.0527 PMC3627420 23150948

[13] - Jaruga A , Hordyjewska E , Kandzierski G , Tylzanowski P . Cleidocranial dysplasia and RUNX2-clinical phenotype–genotype correlation . Clin Genet . 2016 ; 90 ( 5 ): 393 - 402 . doi: 10.1111/cge.12812 10.1111/cge.12812 27272193

[14] - Zhou G , Chen Y , Zhou L , Thirunavukkarasu K , Hecht J , Chitayat D , et al . CBFA1 mutation analysis and functional correlation with phenotypic variability in cleidocranial dysplasia . Human Molecular Genetics . 1999 ; 8 ( 12 ): 2311 - 6 . doi: 10.1093/hmg/8.12.2311 10.1093/hmg/8.12.2311 10545612

[15] - Berkay EG , Elkanova L , Kalayci T , Uludag Alkaya D , Altunoglu U , Cefle K , et al . Skeletal and molecular findings in 51 cleidocranial dysplasia patients from Turkey . Am J Med Genet A . 2021 ; 185 ( 8 ): 2488 - 95 . doi: 10.1002/ajmg.a.62261 10.1002/ajmg.a.62261 33987976

[16] - Quack I , Vonderstrass B , Stock M , Aylsworth AS , Becker A , Brueton L , et al . Mutation analysis of core binding factor A1 in patients with cleidocranial dysplasia . Am J Hum Genet . 1999 ; 65 ( 5 ): 1268 - 78 . doi: 10.1086/302622 10.1086/302622 PMC1288279 10521292

[17] - Hordyjewska-Kowalczyk E , Sowinska-Seidler A , Olech EM , Socha M , Glazar R , Kruczek A , et al . Functional analysis of novel RUNX2 mutations identified in patients with cleidocranial dysplasia . Clin Genet . 2019 ; 96 ( 5 ): 429 - 38 . doi: 10.1111/cge.13610 10.1111/cge.13610 31347140

[18] - Richards S , Aziz N , Bale S , Bick D , Das S , Gastier-Foster J , et al . Standards and guidelines for the interpretation of sequence variants: a joint consensus recommendation of the American College of Medical Genetics and Genomics and the Association for Molecular Pathology . Genet Med . 2015 ; 17 ( 5 ): 405 - 24 . doi: 10.1038/gim.2015.30 10.1038/gim.2015.30 PMC4544753 25741868

[19] - Greulich WW , Pyle SI . Radiographic atlas of skeletal development of the hand and wrist . 2nd ed. Stanford : Stanford University Press ; 1959 . 256 p.

[20] - Cohen MM Jr . Perspectives on RUNX genes: an update . Am J Med Genet Part A . 2009 ; 149A ( 12 ): 2629 - 46 . doi: 10.1002/ajmg.a.33021 10.1002/ajmg.a.33021 19830829

[21] - Zeng L , Wei J , Han D , Liu H , Liu Y , Zhao N , et al . Functional analysis of novel RUNX2 mutations in cleidocranial dysplasia . Mutagenesis . 2017 ; 32 ( 4 ): 437 - 43 . doi: 10.1093/mutage/gex012 10.1093/mutage/gex012 28505335

[22] - Xuan D , Li S , Zhang X , Hu F , Lin L , Wang C , et al . Mutations in the RUNX2 gene in Chinese patients with cleidocranial dysplasia . Ann Clin Lab Sci . 2008 ; 38 ( 1 ): 15 - 24 . 18316777

[23] - Singh A , Goswami M , Pradhan G , Han M-S , Choi J-Y , Kapoor S . Cleidocranial dysplasia with normal clavicles: a report of a novel genotype and a review of seven previous cases . Mol Syndromol . 2015 ; 6 ( 2 ): 83 - 6 . doi: 10.1159/000375354 10.1159/000375354 PMC4521062 26279653

[24] - Medina O , Muñoz N , Moneriz C . Displasia cleidocraneal: reporte de un caso [ Cleidocranial dysplasia: a case report ]. Rev Chil Pediatr . 2017 ; 88 ( 4 ): 517 - 23 . Spanish . doi: 10.4067/S0370-41062017000400012 10.4067/S0370-41062017000400012 28898321

[25] - Suda N , Hattori M , Kosaki K , Banshodani A , Kozai K , Tanimoto K , et al . Correlation between genotype and supernumerary tooth formation in cleidocranial dysplasia . Orthod Craniofac Res . 2010 ; 13 ( 4 ): 197 - 202 . doi: 10.1111/j.1601-6343.2010.01495.x 10.1111/j.1601-6343.2010.01495.x 21040462

[26] - Bergwitz C , Prochnau A , Mayr B , Kramer FJ , Rittierodt M , Berten HL , et al . Identification of novel CBFA1/RUNX2 mutations causing cleidocranial dysplasia . J Inherit Metab Dis . 2001 ; 24 ( 6 ): 648 - 56 . doi: 10.1023/a:1012758925617 10.1023/a:1012758925617 11768584

[27] - Chen S , Gluhak-Heinrich J , Wang YH , Wu YM , Chuang HH , Chen L , et al . Runx2, osx, and dspp in tooth development . J Dent Res . 2009 ; 88 ( 10 ): 904 - 9 . doi: 10.1177/0022034509342873 10.1177/0022034509342873 PMC3045537 19783797

[28] - Camilleri S , McDonald F . Runx2 and dental development . Eur J Oral Sci . 2006 ; 114 ( 5 ): 361 - 73 . doi: 10.1111/j.1600-0722.2006.00399.x 10.1111/j.1600-0722.2006.00399.x 17026500

[29] - D’Souza RN , Aberg T , Gaikwad J , Cavender A , Owen M , Karsenty G , et al . Cbfa1 is required for epithelial-mesenchymal interactions regulating tooth development in mice . Development . 1999 ; 126 ( 13 ): 2911 - 20 . doi: 10.1242/dev.126.13.2911 10.1242/dev.126.13.2911 10357935

[30] - Wang XP , Aberg T , James MJ , Levanon D , Groner Y , Thesleff I . Runx2 (Cbfa1) inhibits Shh signaling in the lower but not upper molars of mouse embryos and prevents the budding of putative successional teeth . J Dent Res . 2005 ; 84 ( 2 ): 138 - 43 . doi: 10.1177/154405910508400206 10.1177/154405910508400206 15668330

[31] - Jensen BL , Kreiborg S . Development of the dentition in cleidocranial dysplasia . J Oral Pathol Med . 1990 ; 19 ( 2 ): 89 - 93 . doi: 10.1111/j.1600-0714.1990.tb00803.x 10.1111/j.1600-0714.1990.tb00803.x 2341976

[32] - Verma R , Jindal MK , Maheshwari S . Familial Cleidocranial Dysplasia . Int J Clin Pediatr Dent . 2010 ; 3 ( 1 ): 57 - 61 . doi: 10.5005/jp-journals-10005-1055 10.5005/jp-journals-10005-1055 PMC4955046 27625558

[33] - Singhal P , Singhal A , Jayam C , Bandlapalli A . Cleidocranial dysplasia syndrome (CCD) with an unusual finding in a young patient . BMJ Case Reports . 2015 : bcr2015210514 . doi: 10.1136/bcr-2015-210514 10.1136/bcr-2015-210514 PMC4654131 26581700

[34] - Sakai N , Hasegawa H , Yamazaki Y , Ui K , Tokunaga K , Hirose R , et al . A case of a Japanese patient with cleidocranial dysplasia possessing a mutation of CBFA1 gene . J Craniofac Surg . 2002 ; 13 ( 1 ): 31 - 4 . doi: 10.1097/00001665-200201000-00005 10.1097/00001665-200201000-00005 11886988

[35] - Moffatt P , Ben Amor M , Glorieux FH , Roschger P , Klaushofer K , Schwartzentruber JA , et al . Metaphyseal dysplasia with maxillary hypoplasia and brachydactyly is caused by a duplication in RUNX2 . Am J Hum Genet . 2013 ; 92 ( 2 ): 252 - 8 . doi: 10.1016/j.ajhg.2012.12.001 10.1016/j.ajhg.2012.12.001 PMC3567275 23290074

[36] - Al-Yassin A , Calder AD , Harrison M , Lester T , Lord H , Oldridge M , et al . A three-generation family with metaphyseal dysplasia, maxillary hypoplasia and brachydactyly (MDMHB) due to intragenic RUNX2 duplication . Eur J Hum Genet . 2018 ; 26 ( 9 ): 1288 - 93 . doi: 10.1038/s41431-018-0166-7 10.1038/s41431-018-0166-7 PMC6117264 29891876

[37] - Ckvs N , Dinkar AD , Khorate M , Khurana S . Cleidocranial dysplasia-a case report and review of literature . IOSR J Dent Med Sci . 2016 ; 15 ( 12 ): 20 - 5 . doi: 10.9790/0853-1512022025

[38] - Yunis E , Varón H . Cleidocranial dysostosis, severe micrognathism, bilateral absence of thumbs and first metatarsal bone, and distal aphalangia: a new genetic syndrome . Am J Dis Child . 1980 ; 134 ( 7 ): 649 - 53 . doi: 10.1001/archpedi.1980.02130190017005 10.1001/archpedi.1980.02130190017005 7395825

[39] - Biswas A , Gogineni S , Rao K , Sakthivel S , Castelino R . Application of 3-D imaging in a familial case of cleidocranial dysplasia . Cumhuriyet Dent J . 2020 ; 23 ( 2 ): 142 - 8 . doi: 10.7126/cumudj.668128

