# Supplementary material for: Cleidocranial dysplasia and novel RUNX2 variants: dental, craniofacial, and osseous manifestations
Source: J Appl Oral Sci. 2022 Jun 6;30:e20220028. doi: 10.1590/1678-7757-2022-0028 (PMC9239300; doi:10.1590/1678-7757-2022-0028)
Supplement: Supplementary file 1 [file 1678-7757-jaos-30-e20220028-supl1.pdf]

Original Article
http://dx.doi.org/10.1590/1678-7757-2022-0028

JAOS
JOURNAL OF APPLIED ORAL SCIENCE

Supplementary Figure 1
References for Table 2

1. Zhang X, Liu Y, Wang X, Sun X, Zhang C, Zheng S. Analysis of novel RUNX2 mutations in Chinese patients with cleidocranial dysplasia. PLoS One. 2017;12(7):e0181653. doi: 10.1371/journal.pone.0181653
2. Quack I, Vonderstrass B, Stock M, Aylsworth AS, Becker A, Brueton L, et al. Mutation analysis of core binding factor A1 in patients with cleidocranial dysplasia. Am J Hum Genet. 1999;65(5):1268-78. doi: 10.1086/302622
3. Gao X, Li K, Fan Y, Sun Y, Luo X, Wang L, et al. Identification of RUNX2 variants associated with cleidocranial dysplasia. Hereditas. 2019;156:31. doi: 10.1186/s41065-019-0107-7
4. Hordyjewska-Kowalczyk E, Sowińska-Seidler A, Olech EM, Socha M, Glazar R, Kruczek A, et al. Functional analysis of novel RUNX2 mutations identified in patients with cleidocranial dysplasia. Clin Genet. 2019;96(5):429-38. doi: 10.1111/cge.13610
5. Baumert U, Golan I, Redlich M, Aknin JJ, Muessig D. Cleidocranial dysplasia: molecular genetic analysis and phenotypic-based description of a Middle European patient group. Am J Med Genet A. 2005;139A(2):78-85. doi: 10.1002/ajmg.a.30927
6. Yoshida T, Kanegane H, Osato M, Yanagida M, Miyawaki T, Ito Y, et al. Functional analysis of RUNX2 mutations in Japanese patients with cleidocranial dysplasia demonstrates novel genotype-phenotype correlations. Am J Hum Genet. 2002;71(4):724-38. doi: 10.1086/342717
7. Tessa A, Salvi S, Casali C, Garavelli L, Digilio MC, Dotti MT, et al. Six novel mutations of the RUNX2 gene in Italian patients with cleidocranial dysplasia. Hum Mutat. 2003;22(1):104. doi: 10.1002/humu.9155
8. Xuan D, Li S, Zhang X, Hu F, Lin L, Wang C, Zhang J. Mutations in the RUNX2 gene in Chinese patients with cleidocranial dysplasia. Ann Clin Lab Sci. 2008;38(1):15-24
9. Ryoo HM, Kang HY, Lee SK, Lee KE, Kim JW. RUNX2 mutations in cleidocranial dysplasia patients. Oral Dis. 2010;16(1):55-60. doi: 10.1111/j.1601-0825.2009.01623.x
10. Sakai N, Hasegawa H, Yamazaki Y, Ui K, Tokunaga K, Hirose R, et al. A case of a Japanese patient with cleidocranial dysplasia possessing a mutation of CBFA1 gene. J Craniofac Surg. 2002;13(1):31-4. doi: 10.1097/00001665-200201000-00005
11. Zeng L, Wei J, Han D, Liu H, Liu Y, Zhao N, et al. Functional analysis of novel RUNX2 mutations in cleidocranial dysplasia. Mutagenesis. 2017;32(4):437-43. doi: 10.1093/mutage/gex012
12. Lee KE, Seymen F, Ko J, Yildirim M, Tuna EB, Gencay K, et al. RUNX2 mutations in cleidocranial dysplasia. Genet Mol Res. 2013;12(4):4567-74. doi: 10.4238/2013.October.15.5
13. Medina O, Muñoz N, Moneriz C. Displasia cleidocraneal: reporte de un caso [Cleidocranial dysplasia: a case report]. Rev Chil Pediatr. 2017;88(4):517-23. Spanish. doi: 10.4067/S0370-41062017000400012
14. Bufalino A, Paranaíba LM, Gouvêa AF, Gueiros LA, Martelli-Júnior H, Junior JJ, et al. Cleidocranial dysplasia: oral features and genetic analysis of 11 patients. Oral Dis. 2012;18(2):184-90. doi: 10.1111/j.1601-0825.2011.01862.x
15. Hansen L, Riis AK, Silahtaroglu A, Hove H, Lauridsen E, Eiberg H, et al. RUNX2 analysis of Danish cleidocranial dysplasia families. Clin Genet. 2011;79(3):254-63. doi: 10.1111/j.1399-0004.2010.01458.x
16. Lin WD, Lin SP, Wang CH, Tsai Y, Chen CP, Tsai FJ. RUNX2 mutations in Taiwanese patients with cleidocranial dysplasia. Genet Mol Biol. 2011;34(2):201-4. doi: 10.1590/s1415-47572011005000002
17. Kim HJ, Nam SH, Kim HJ, Park HS, Ryoo HM, Kim SY, et al. Four novel RUNX2 mutations including a splice donor site result in the cleidocranial dysplasia phenotype. J Cell Physiol. 2006;207(1):114-22. doi: 10.1002/jcp.20552
18. Napierala D, Garcia-Rojas X, Sam K, Wakui K, Chen C, Mendoza-Londono R, et al. Mutations and promoter SNPs in RUNX2, a transcriptional regulator of bone formation. Mol Genet Metab. 2005;86(1-2):257-68. doi: 10.1016/j.ymgme.2005.07.012
19. Jung YJ, Bae HS, Ryoo HM, Baek SH. A novel RUNX2 mutation in exon 8, G462X, in a patient with cleidocranial dysplasia. J Cell Biochem. 2018;119(1):1152-62. doi: 10.1002/jcb.26283
20. Zhou G, Chen Y, Zhou L, Thirunavukkarasu K, Hecht J, Chitayat D, et al. CBFA1 mutation analysis and functional correlation with phenotypic variability in cleidocranial dysplasia. Hum Mol Genet. 1999;8(12):2311-6. doi: 10.1093/hmg/8.12.2311
