# Supplementary material for: Cleidocranial dysplasia and novel RUNX2 variants: dental, craniofacial, and osseous manifestations
Source: J Appl Oral Sci. 2022 Jun 6;30:e20220028. doi: 10.1590/1678-7757-2022-0028 (PMC9239300; doi:10.1590/1678-7757-2022-0028)
Supplement: Supplementary file 2 [file 1678-7757-jaos-30-e20220028-supl2.pdf]

Original Article
http://dx.doi.org/10.1590/1678-7757-2022-0028

JOURNAL OF APPLIED ORAL SCIENCE

Supplementary Table 1
Primers for Sanger sequencing

Families	Sense Sequence	Antisense Sequence
1	TACCCTCTGCTTATGGGCCT	CAGACCTTGTTCTTGGATATTACGA
2	TCTAAGGCTGCAATGGTTGCT	ACTGTGAGCATGGATGAGACA
3	TGGCCACCAGATACCGCTTA	ATAAGCCGCTTCACAGCTCC
4	CCTAAAGTGGTCATCGGAGGG	ATTTCCCAAAGCTACGGAGGC
5	CCTAAAGTGGTCATCGGAGGG	ATTTCCCAAAGCTACGGAGGC
